# Tumor-Cell–Macrophage Fusion Cells as Liquid Biomarkers and Tumor Enhancers in Cancer

**DOI:** 10.3390/ijms21051872

**Published:** 2020-03-09

**Authors:** Yariswamy Manjunath, David Porciani, Jonathan B. Mitchem, Kanve N. Suvilesh, Diego M. Avella, Eric T. Kimchi, Kevin F. Staveley-O’Carroll, Donald H. Burke, Guangfu Li, Jussuf T. Kaifi

**Affiliations:** 1 Department of Surgery, Ellis Fischel Cancer Center, University of Missouri, Columbia, MO 65212, USA; yariswamym@health.missouri.edu (Y.M.); mitchemj@health.missouri.edu (J.B.M.); suvileshk@missouri.edu (K.N.S.); avellapatinod@health.missouri.edu (D.M.A.); kimchie@health.missouri.edu (E.T.K.); ocarrollk@health.missouri.edu (K.F.S.-O.); liguan@health.missouri.edu (G.L.); 2 Harry S. Truman Memorial Veterans’ Hospital, Columbia, MO 65201, USA; 3 Department of Molecular Microbiology & Immunology, University of Missouri, Columbia, MO 65212, USA; porcianid@missouri.edu (D.P.); burkedh@missouri.edu (D.H.B.); 4 Bond Life Sciences Center, University of Missouri, Columbia, MO 65212, USA; 5 Department of Biochemistry, University of Missouri, Columbia, MO 65212, USA

**Keywords:** cancer, liquid biomarkers, circulating tumor cells, fusion cells

## Abstract

Although molecular mechanisms driving tumor progression have been extensively studied, the biological nature of the various populations of circulating tumor cells (CTCs) within the blood is still not well understood. Tumor cell fusion with immune cells is a longstanding hypothesis that has caught more attention in recent times. Specifically, fusion of tumor cells with macrophages might lead to the development of metastasis by acquiring features such as genetic and epigenetic heterogeneity, chemotherapeutic resistance, and immune tolerance. In addition to the traditional FDA-approved definition of a CTC (CD45-, EpCAM+, cytokeratins 8+, 18+ or 19+, with a DAPI+ nucleus), an additional circulating cell population has been identified as being potential fusions cells, characterized by distinct, large, polymorphonuclear cancer-associated cells with a dual epithelial and macrophage/myeloid phenotype. Artificial fusion of tumor cells with macrophages leads to migratory, invasive, and metastatic phenotypes. Further studies might investigate whether these have a potential impact on the immune response towards the cancer. In this review, the background, evidence, and potential relevance of tumor cell fusions with macrophages is discussed, along with the potential role of intercellular connections in their formation. Such fusion cells could be a key component in cancer metastasis, and therefore, evolve as a diagnostic and therapeutic target in cancer precision medicine.

## 1. Introduction

Cancer progression and metastasis are highly complex processes consisting of significant molecular changes [1]. Circulating tumor cells (CTCs) are cancer cells that are shed from a primary tumor into the vasculature, circulate throughout the body, have the potential to settle in distant organs and develop into solid organ tumor metastases that are responsible for most cancer-associated deaths [2]. Detection of CTCs in the blood via a simple and minimally-invasive venous blood draw as a form of a ‘liquid biopsy’ has significant benefits over invasive tissue biopsies. Invasive tissue biopsies have significant risks; however, are still the standard for treatment planning in cancer patients. In addition to potential complexity, high cost, and single time-point limitations of the typical tissue biopsy, treatment responses, and monitoring are exclusively based on costly and indirect image interpretation that includes radiation exposure to the patient. To address these risks and limitations, liquid biomarkers such as CTCs detected via peripheral blood draws are a unique opportunity to characterize tumor biology and personalize clinical treatment decisions by analyzing whole tumor cells in real-time. The FDA approved the CellSearch^®^ system in 2007 for immunoaffinity-based CTC detection and enumeration in 7.5 mL of peripheral whole blood from metastatic breast and colorectal cancer patients. Since then, a CTC has been defined as having a well-defined 4′,6-diamidino-2-phenylindole (DAPI)+ nucleus that expresses cytokeratin (CK) 8/18 and/or 19 and EpCAM (epithelial cell markers), but distinctively lacks the expression of CD45 (a pan-leukocyte marker) [3]. This rather broad definition has been shown to be incomplete as potentially highly relevant circulating cancer-associated cells might lack EpCAM, or even co-express CD45 and other macrophage/myeloid or stem-cell markers [4,5,6,7,8] (Table 1). The provocative hypothesis that tumor cells fuse with white blood cells to migrate and escape the immune surveillance to grow to metastases in other organs, was developed more than 100 years ago [9]. Recently several groups have proposed cancer cell fusions, and in particular leukocyte-tumor cell fusions, to have potential significant roles in tumor progression, cancer metastasis, and chemotherapy resistance in solid cancers [10,11]. Molecular mechanisms of cellular fusion have been suggested, and intercellular connections (e. g., via tunneling nanotubes (TNTs)) might play a role in partial and permanent cell fusions [12,13]. Experimental results indicate that tumor cell fusions with other tumor cells (homotypic) or with non-tumorous cell types (heterotypic) are associated with more aggressive cancer phenotypes (such as patient survival, metastatic capabilities, and chemoresistance) [14]. Recent reports on circulating cancer-associated cells with dual epithelial and macrophage/myeloid phenotypes, combined with genetic analyses, have supported the idea that fusion events between tumor cells and macrophages might have a critical role in tumor progression, development of metastatic disease, and poor outcome for cancer patients [12,15,16,17,18,19,20,21,22]. Beyond the potential clinical utility of these fusion cells as blood-borne liquid biomarkers, via a simple phlebotomy in precision oncology, further scientific exploration of fusion molecular mechanisms and the impact of fusion cells could fundamentally advance our understanding of metastatic cancer biology and lead to the identification of novel therapeutic strategies.

## 2. Cell Fusion as a Physiological and Pathological Mechanism

Cell fusion is observed in various physiological processes and pathological conditions. A notable benefit of artificially generated hybrid cells (‘hybridoma’) has been gained by artificially fusing myeloma cells with lymphocytes to produce monoclonal antibodies in large quantities [30]. However, cell fusion is fundamental in multiple developmental and biological processes, such as fertilization, placentation, myogenesis, osteogenesis, wound healing, and tissue regeneration. Fusion has been described as ‘homotypic’ when it is between two cells of the same cell types (e.g., between two myoblasts, two trophoblasts [31], two macrophages [31,32], etc.), and as ‘heterotypic’ when it is between different cell types (e.g., amongst gametes [31] and tumor cells with various other cell types [12,15,33,34]). In pathological non-cancerous conditions (such as granulomata from infections (mycobacteria, viral (herpes, HIV)), foreign body reactions), heterotypic fusion of cells of myeloid/monocyte/macrophage lineage leads to multinucleated giant cells [32]. These are described as foreign-body giant cells, osteoclasts, Langhans giant cells, Touton giant cells, giant cells in temporal arteritis, or Reed–Sternberg cells in Hodgkin’s lymphoma.

Using model organism studies it has been suggested that there is a genetic program for non-pathological, physiological cell fusion that is separated in three stages—(1) cell induction and differentiation (competence), (2) cell determination, migration, and adhesion (commitment), and (3) membrane merging and cytoplasmic mixing (cell fusion) [31]. Fusogens are cellular proteins that have been identified to mediate fusion of cell membranes. For instance, Syncytins belong to a fusogen family that contains diverse proteins that originated from endogenous retroviruses related to the HIV gp41 envelope glycoprotein, and they play a role in the formation of syncytial trophoblasts in mouse placentation [35]. Then, the F protein family plays a role in cell fusions in the cutaneous, gastrointestinal, and reproductive organs of the nematode *Caenorhabditis elegans* [36]. Fusogens in human cells, and in particular in tumor cells, still needs to be identified, to further understand the genetic and biological mechanisms of cancer cell fusions with themselves and other cell types.

Tumor cell fusions have also been found to occur homotypically with other tumor cells [37,38], but also heterotypically with fibroblasts [14,39], stem cells [40], and myeloid-derived cells [15,28,41]. Different techniques have been developed to induce artificial cellular fusion for experimental purposes. These include electrofusion (causing hydrophilic pores in the membrane lipid bilayer through electroporation, leading to fusion) [42], incubation with polyethylene glycol (PEG) (causing redistribution of intramembranous particles of cellular membranes, leading to fusion with little cellular toxicity) [43], or induction with the *Sendai* virus (also called the hemagglutination virus of Japan (HVJ)), which has been used to generate hybridomas, to make monoclonal antibodies [30]. 

The molecular mechanisms of cell fusion processes are not well defined or understood. The interaction of CD40 and CD40L between CD4+ T lymphocytes and monocytes results in T cell activation and in interferon (IFN)-γ secretion, which subsequently leads to secretion of a fusion-related molecule—dendritic cell-specific transmembrane protein (DC-STAMP)—by monocytes, resulting in the formation of Langhans giant cells [44]. Additionally, apoptosis and pro-inflammatory cytokines, such as the tumor necrosis factor (TNF)-α, have been shown to promote cell fusions [13]. Fusion between mesenchymal/multipotent stem cells and breast tumor cells is significantly increased under hypoxic conditions, with the apoptotic neighboring cells leading to enhanced fusion [13]. Apoptotic cells can promote fusion of myoblasts, an observation that is linked to the signaling process via the phosphatidylserine receptor brain specific angiogenesis inhibitor 1 (BAI1) pathway [45]. BAI1 triggers the internalization of apoptotic cells with the ELMO/Dock180/Rac signaling segment. ELMO and Dock180 are combined guanine nucleotide exchange factors for the GTPase Rac, and they regulate the actin-mediated cytoskeleton changes necessary for phagocytosis of apoptotic cell fragments [46]. Myoblasts and macrophages mediate their fusions via a similar molecular mechanism [47]. As expected, the cytoskeleton plays a key role in cell fusion, and studies in *Drosophila* flies have demonstrated membranous juxtaposition and cell fusion that is driven by the mechanical tension of cell membranes via a non-muscle Myosin II-mediated mechanosensory response to the invasive force from the partnering fusion cell [48]. It is not yet known whether tumor cells use similar molecular mechanisms for homo- and heterotypic cell fusion. 

It is well-known that various cell types spontaneously form homo- and heterotypical fusions in co-culture in vitro. Spontaneous fusion was observed in vitro between breast tumor cells themselves [37], but also between breast tumor cells and other cells (e.g., normal breast epithelium [49], endothelial [50], stromal cells, and stem cells [13,51]). Heterotypic fusions between tumor cells and stem cells, in addition to other cell types, have been specifically suggested to contribute to tumor progression [13]. In xenograft experiments in non-obese diabetic–severe combined immunodeficient (NOD/SCID) mice, fusion was described between human lung tumor cell line cells and bone marrow-derived mesenchymal stem cells [51]. Breast tumor cells can spontaneously fuse with mesenchymal stem cells to form hybrid cells that have increased invasion and migratory capacity, which is clearly a cancer-promoting feature [13]. After fusion of human hepatocellular carcinoma cells with mesenchymal stem cells, these hybrid cells have a higher metastatic potential in mice than the non-fused hepatocellular carcinoma parental cells [52].

In addition to fusion between tumor cells and macrophages, it appears that other heterotypic fusion events clearly also need to be further explored to understand metastatic cancer biology. However, promising experimental pilot data have been developed that deserve further molecular investigation to understand the cancer biology of tumor cell/macrophage fusions.

## 3. Genetic Evidence for Presence of Fusion Cells in Cancer Patients

Few data exist on the presence of fusion cells in the primary cancer tissue, and it is unknown which compartment fusion between tumor and other cells types occur in cancer patients (primary tumor site, peritumoral microvessels, intravascular, lymphatic system, bone marrow, etc.) [16]. The most convincing proof of the existence of fusion cells in human cancers has been the demonstration of hybrid genomes in sex-mismatch bone marrow transplantation recipients that developed cancers later on [17,18,53]. Donor DNA was found in a pediatric patient that developed a renal cell carcinoma, after bone marrow transplantation [54]. In another genetic analysis of a female recipient of a sex-mismatch bone marrow transplant who also developed a renal cell carcinoma, the Y chromosome of the male donor and a trisomy of chromosome 17—consistent with the trisomy found in the primary renal cell carcinoma and the Y chromosome from the male donor—were co-located presumably in tumor cells [53]. In human melanoma tissue samples, cells carrying the BRAF^V600E^ mutation were found among cells surrounding the primary tumor, in the stroma of melanoma metastases, and in melanoma cells of a local recurrence re-excision specimen [39]. These findings were most likely due to a heterotypic fusion of melanoma cells with other cells, as these BRAF^V600E^ mutations were present in peritumoral Melanoma Antigen Recognized by T cells (MART1)+/smooth muscle antigen (SMA)+ fibroblasts and MART1+/CD68+ macrophages [39]. In addition to primary melanoma tissue samples, these peritumoral stromal cells carrying the melanoma-derived BRAF^V600E^ mutations were also identified in melanoma metastases, implicating a role of these fusion cells in the cancer spread [39]. Then, the circulating macrophage-melanoma fusion cells in patient blood morphologically and ultrastructurally looked like macrophages in electron microscopy, and they carried highly abnormal DNA contents with melanoma-specific mutations in the BRAF gene, consistent with the mutations found in the corresponding primary melanomas [22]. In another study, tumor tissue specimens from seven female cancer patients who had received sex-mismatched bone marrow transplants and later on developed solid cancers (renal cell carcinoma, head and neck squamous cell carcinoma, and lung adenocarcinoma) contained evidence of tumor cell fusion in the form of CK+ epithelial tumor cells with Y chromosomes in the nuclei [55]. Another report applied DNA short tandem repeat length polymorphism analysis, demonstrating that a melanoma brain metastasis contained tumor cells that were the result of fusion between melanoma cells and bone marrow transplant-derived cells [17]. Taken together, there is significant genetic evidence supporting the existence of fusion cells in cancer patients.

## 4. Fusion of Tumor Cells with Macrophages

**Figure 1:**
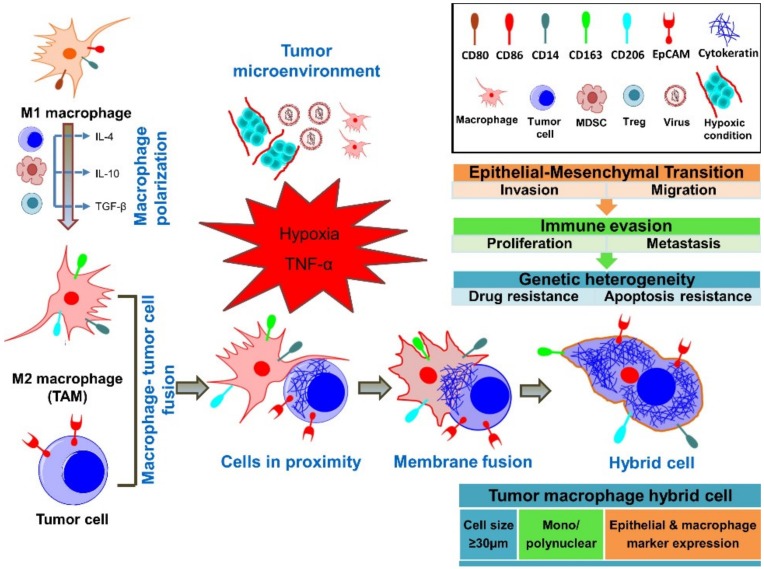
Concepts of fusion between tumor cells and macrophages. It is hypothesized that tumor-associated M2-polarized macrophages (TAMs) fuse their membranes with tumor cells, forming a tumor–macrophage hybrid cell. These fusion cells are large, mononuclear/polynuclear, and express both epithelial and myeloid markers. Importantly, fusion cells exert pro-tumorigenic and pro-metastatic effects through the outlined mechanisms.

Recent reports on circulating cancer-associated cells with both epithelial and macrophage/myeloid phenotypes in cancer patients, combined with genetic evidence, have supported the idea that fusion has a critical role in cancer progression (Figure 1) [12,15,16,56]. Macrophage M1 or M2 polarization appears to be critical for various aspects of immune responses to cancer and its progression [57]. Macrophage infiltration of the primary tumor and polarization depend on cytokines in the tumor microenvironment (TME) [58]. Within the TME, tumor-associated macrophage polarization to the M1 phenotype can be triggered through bacterial lipopolysaccharide (LPS) and by T helper 1 (Th1) cytokines, such as IFN-γ and also by TNF-α [59]. The M1 phenotype is associated with anti-tumor properties [60]. M2 phenotype macrophages have pro-tumoral effects, leading to increased cancer cell survival, proliferation, invasiveness, and immunosuppression in favor of the tumor [57]. M2 polarization is induced by T helper 2 cytokines interleukin (IL)-4, IL-13, macrophage colony-stimulating factor (M-CSF), and transforming growth factor (TGF)-β [60]. M2 macrophages are anti-inflammatory, immunosuppressive, and promote cancer progression, chemoresistance, and metastasis [61,62]. M2 macrophages have critical interactions with tumor cells, but also with cells associated with tumor progression, such as Th2 cells, cancer-associated fibroblasts, regulatory T cells (Tregs), and myeloid-derived suppressor cells [57]. M2 polarization phenotypes have also been observed in tumor fusion cells [63]. Importantly, macrophages also have a high fusogenic potential, which is also likely to occur with tumor cells [18,20,58,64]. In vitro and in vivo studies suggest that metastatic cells can be the result of the fusion of tumor cells with cells of hematopoietic/myeloid lineage, specifically with macrophages [17,18,19,20,21,22,34,54]. Importantly, patient-derived tumor-macrophage fusion cells were shown to have M2 macrophage phenotypes [21,22]. In a murine melanoma metastasis model, certain clones of lung metastasis cells had properties of melanoma cell—macrophage fusion cells [65]. Importantly, fusion of tumor cells with macrophages is supported through the observation of these fusion cells in cancer patients [21,22,27,28]. Macrophage fusion receptor DAP12 expression is associated with higher metastatic rates in breast cancer patients [20,66,67]. It remains unclear whether tumor-associated macrophages fuse within the tumor microenvironment at the site of the tumor, in the blood, or in the lymphatic system. Understanding molecular fusion mechanisms between macrophages and tumor cells and the impact that fusion cells have on the immune system is of high interest in identifying therapeutic targets.

## 5. Fusion Cell Detection in the Peripheral Blood of Cancer Patients

Numerous techniques have been developed for the identification of CTCs in peripheral human blood [5]. Most of these detection platforms rely on the tumor cell expression of epithelial surface markers or on their biophysical characteristics, such as size or density [27,68]. CellSearch^®^ has established clinically prognostic value in a variety of cancers, yet it does not detect and even excludes certain circulating cancer-associated cells [69]. Other CTC isolation techniques (e.g., microfilters based on size [70]) allow additional phenotypic analysis and identification of other potentially relevant circulating cancer-associated cells, including cells that have undergone epithelial–mesenchymal transition (EMT) and down-regulated EpCAM, or which express myeloid/macrophage-markers CD45/14+ [4,5,8,27,28,71,72]. 

In different solid cancers, we and other groups have identified a distinct circulating cancer-associated cell that is large, polymorphic in shape, often polynuclear (≥1 DAPI+ nucleus), with a dual epithelial and macrophage/myeloid phenotype (dual CK+/EpCAM+ and CD14/CD45+) (Table 2) [5,27,28,73,74]. Other groups refer to these likely identical cells as tumor-macrophage fusion cells (TMFs), macrophage-tumor cell fusion cells (MTFs), or cancer-associated macrophage-like (CAMLs) cells [8,27,28,29,73]. While some investigators attributed these cells with equivalent and similar features to be cellular fusion products between tumor cells and macrophages (MTFs), other groups described these large cells in a rather broader way as CAMLs, not clearly hypothesizing on the cellular fusion events but rather describing giant macrophages that contain phagocytosed tumor debris [15,21,22,27,29,73,75,76]. Based on the multiple studies that are discussed in this review, the authors believe that these cells are all identical and are actually a product of tumor cell and macrophage fusion events. In studies by our own group, we enriched large (≥30 µm diameter), polymorphic, mononuclear or polynuclear (syn- or heterokaryon), with microfilters or gradient centrifugation techniques from the peripheral blood of melanoma, pancreatic ductal adenocarcinoma, and colorectal cancer patients [27]. These hybrid cells from patients’ blood could be grown through multiple passages and grown into solid tumors in xenograft models [5,21]. In addition to CD14/45, other cell biomarkers that have been used to imply heterotypic fusion of CTCs are hematopoietic or myeloid/macrophage lineage receptors CD68 or CD163 [5,20,21,22,27,28,39,73]. In these fusion cells, the CK pattern appears to have a rather diffuse cytoplasmic distribution, as described in CTCs with a mesenchymal phenotype, in contrast to the typical filamentous cytoplasmic CK orientation typically found in ‘traditional’ CK+/CD45- CTCs [73]. The detection frequency of macrophage/myeloid tumor fusion cells in the blood of cancers patients can be >40%, depending on the detection techniques used [16,20,53,55,77,78]. Analyzed patients were suffering from breast cancer [20,66], colorectal cancer [21,28,77], pancreatic ductal adenocarcinoma [21,55], ovarian cancer [16], renal cell carcinoma [53,54], head and neck squamous cell carcinoma [55], non-small cell lung cancer [55], malignant melanoma [17,18,39], or multiple myeloma [78]. In pancreatic ductal adenocarcinoma, presence of these fusion cells was associated with a higher cancer stage and poorer survival [55]. Metastatic breast cancer patients with macrophage/tumor cell fusions in the blood have higher cancer stages and worse clinical outcomes, when measured in terms of progression-free and overall survival [79]. In a cohort of 269 patients with solid cancers of different origins (breast, prostate, pancreatic, lung, kidney, and esophagus) and clinical stages, an association with poor survival was shown if ≥6 fusion cells were present in 7.5 mL of whole blood or if fusion cells were ≥50 µm in size [76]. Importantly, in a study of the same group, a high prevalence of fusion cells in the blood of early-stage cancer patients was observed, making them a potential liquid biomarker for early cancer detection in screening settings [29,73]. The authors also described the large and heterogeneous cell morphologies and distinct marker expression staining patterns for CK and CD14/45 of these cells, in detail [29,73]. These interesting findings on the negative impact of tumor cell/macrophage fusion cells on cancer patients’ outcome suggest a highly-informative correlation of fusion cells with aggressive tumor biology in humans.

## 6. Cancer-Associated Properties of Fusion Cells

Cancer progression and metastasis is a highly complex process consisting of various molecular changes. A critical component in cancer progression is epithelial–mesenchymal transition (EMT), during which tumor cells lose intercellular adhesions, downregulate epithelial molecules (e.g., EpCAM), and up-regulate the expression of mesenchymal molecules, such as N-cadherin and vimentin [80]. EMT leads to migratory and invasive properties of tumor cells, which allows detachment from the primary cancer, to migrate and form metastases in distant organs [6]. Later on, those hybrids transition back to epithelium-like morphology, consistent with the process of reverse EMT, which is described as a mesenchymal–epithelial transition (MET) of tumor cells [81]. The detachment of tumor cells from the primary tumor, intravasation, and transit in the bloodstream and extravasation into distant organ sites requires a complex adaptation to new microenvironments with subsequent cellular proliferation to generate a metastatic cancer nodule [82]. The idea of tumor cells acquiring mobility by fusion with naturally migratory macrophages appears to be an attractive theory (Table 3) [83]. Obviously, the blood vasculature is a liquid biopsy compartment to detect fusion cells in cancer patients with a localized tumor and no evidence of distant metastases on staging imaging. Therefore, fusion cells should be detectable in early-stage cancer patients as a micrometastatic (or minimal residual) disease, even before any solid organ metastases are present [2]. One study demonstrated in melanoma patients with no radiographic evidence of distant metastases that in histologically negative lymph nodes, unique macrophage-like cells expressed mRNA for Melanoma Antigen Recognized by T cells 1 (MART-1) and contained melanin granules [84]. The authors suggested that some of these cells might represent macrophage-melanoma hybrids, such as a CD68 protein that is co-expressed with MART-1 mRNA [84].

A cell motility gene signature that included p38 mitogen-activated protein kinase (p38 MAPK) signaling pathways (known to be involved in cancer cell migration) has been described in CTCs derived from pancreatic ductal adenocarcinoma patients [92]. Another study also indicated that fusion between tumor cells of ovarian and lung origin and myeloid lineage cells leads to a significantly higher expression of chemokine receptor CXCR4, which is associated with the migration of tumor cells to the bone marrow [16,93]. The enhanced migration capability of in vitro generated breast cancer tumor and normal cell fusions is also linked to chemokine receptor CCR7 and a ligand CCL21 interaction that is associated with the migration of breast cancer cells to the lymph nodes [49,85]. Increased migratory and metastatic potential of fusion cells was also observed in vivo when human breast tumor cell lines injected in nude mice formed hybrids that metastasized to the lungs and bones [37]. The majority of hybrids produced by in vitro fusion of macrophages with melanoma cells showed enhanced metastatic potential in vivo in mice, increased motility in vitro, increased ability to produce melanin, and higher responsiveness to melanocyte stimulating hormone (MSH), compared to non-fused melanoma cells [65]. The metastatic potential of fused macrophages and melanoma cells in vitro correlated with increased expression of malignancy-associated GnT-V and β1,6-branching in glycoproteins, which has been previously described in tumor associated macrophages [94].

In general, in vitro tumor cell fusion study results need to be interpreted with caution as there might potentially be significant differences in the expression and cellular behavior patterns between spontaneous fusions or artificial PEG-induced fusions. A recent study demonstrated that artificial fusion between breast tumor cells and macrophages using PEG promotes proliferation, migration, invasion, and colony formation of breast tumor cells by activating the tumor-promoting Wnt/β-catenin signaling pathway [87]. These fusion cells displayed EMT with a significant downregulation of E-cadherin and up-regulation of N-cadherin, vimentin, and snail, as well as an increased expression of MMP-2, MMP-9, uPA, and S100A4 [87]. Mechanistically, the TCF/LEF transcription factor activity of the Wnt/β-catenin pathway and downstream target genes, including cyclin D1 and c-Myc, were increased in the fusion cells [87]. Proliferation, migration, and invasion caused by fusion could be blocked by treatment with XAV-939, a Wnt/β-catenin signaling pathway inhibitor [87]. At the site of the primary tumor, macrophage migration inhibitory factor (MIF) can induce EMT and tumorigenicity in many cancer types [95]. MIF has a role in M2 polarization of macrophages, and MIF has been shown to be expressed by melanoma macrophage fusion cells derived from patients [22].

Tumorigenicity of patient-derived tumor fusion cells is demonstrated by their ability to form tumors in murine xenograft experiments. In vitro/vivo expansions of CTCs are very challenging and rarely succeed beyond the brief culturing time and rarely convert to a stable cell line or CTC-derived xenograft tumor models (CDX) [96]. Even culturing of CTCs from widely metastatic breast cancer patients carrying large numbers of CTCs were unsuccessful to form metastatic foci in NOD/SCID mice [97]. However, using gradient centrifugation techniques, followed by xenografting, cultures of macrophage/tumor cell fusion cells from the blood of melanoma and pancreatic ductal adenocarcinoma patients led to dissemination and metastatic lesions in distant organ sites of nude mice [21,22]. These cells expressed both primary melanocytic tumor markers (MLANA, ALCAM) and tumor-promoting M2 macrophage markers (CD206, CD208) [21,22]. Despite the limitations of circulating cancer-associated fusion cell clonal expansions in the culture and xenografting, patient-derived models will need to be further explored to shed light on the biological and clinical impact of tumor cell fusions.

## 7. Intercellular Connections between Tumor Cells and Macrophages Lead to Partial Cell Fusion, and Favor Cell Migration and Invasion

**Figure 2:**
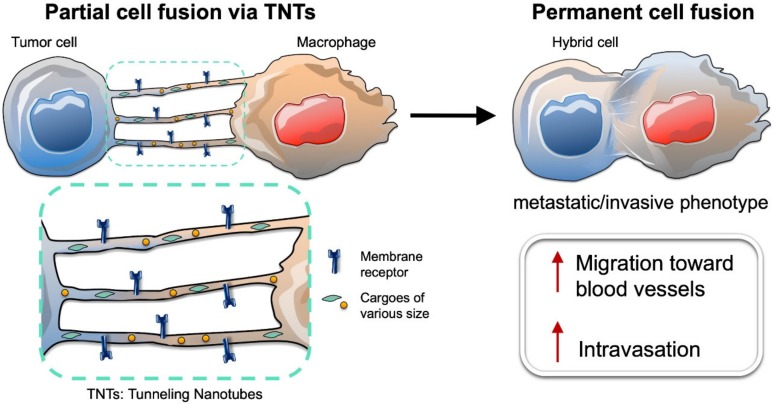
Illustration of partial cell fusion via tunneling nanotubes (TNTs) and permanent cell fusion. (up-arrow: increased capacity).

Permanent cell fusion between tumor cells and macrophages represents the final stage of a transformative biological process of progressively evolving cell-to-cell interactions (Figure 2). In the highly hypoxic and acidic milieu of the tumor microenvironment, intercellular communication between tumor cells and stromal cells (including macrophages) is achieved by paracrine signaling that exploits secreted signaling molecules and exosomes, or by juxtacrine signaling that instead uses transient cell-to-cell contacts (e.g., gap junctions or membrane protrusions) [98,99]. Interestingly, cell-to-cell communication mechanisms that exploit membrane protrusions were only discovered in recent years [100,101]. These protrusions enable exchange of specific signals by direct cell-to-cell contact over short (tens of microns) and long (hundreds of microns) distances. Thin and transient membrane protrusions have been found, both in vitro [102,103,104] and in vivo [105,106,107] in different tissues from various organisms and in several disease models. Each protrusion type possesses distinct structural and functional characteristics. Among them, filopodia-derived protrusions, such as cytonemes, and Tunneling NanoTubes (TNTs), are the most studied [108,109]. Several recent reviews have investigated the biological properties and structural characteristics of these thin membrane protrusions and their roles in developmental biology and pathogenesis (including cancers and infectious diseases) [98,110,111,112]. In this section, we focus mostly on TNTs (open-ended cellular projections that display diameters ranging from 20 to 500 nm) and their role in tumor cell interactions with macrophages (Figure 2).

TNTs are membrane protrusions typically composed of filamentous (F)-actin, and to a lesser extent, of microtubules [110,113]. They allow a direct physical connection between the cytoplasms of two or more cells that belong to different cell types (heterotypic) or the same cell type (homotypic), in addition to establishing continuity of their plasma membranes. Notably, formation of TNTs lead to “partial cell fusion” [114], which enables the mobilization and exchange of cargoes of various sizes—ions (e.g., calcium) [115], molecules (e.g., membrane receptors and signaling proteins) [102], vesicles (e.g., endosomes or lysosomes) [102], and even organelles (e.g., mitochondria) [116]. TNTs that connect the cytoplasms of two cells are named “open-ended TNTs” [98,110]. In addition, “close-ended TNTs” have also been identified, although it is still unclear whether they represent an intermediate status in the process of open-ended TNT formation or if instead the signaling is transported using a different mechanism (such as synapse-like mechanisms). For instance, it has been demonstrated that transfer of viral HIV-1 particles through close-ended TNTs from infected to uninfected T cells was dependent on the interaction between viral protein Env and its host receptor CD4 at the tip of the TNTs [117]. This mechanism is known as the virological synapse.

TNTs can be formed between previously unconnected cells through an extension of filopodia-like protrusions. This mechanism is known as “protrusion elongation” [100,110] and involves actin polymerization factors, including the Rho GTPase family proteins Rac1 and Cdc42 [110,118]. Alternatively, formation of TNTs can occur by dislodgement of two initially attached cells in which short filopodia-like protrusions enable direct cytoplasm connection. Upon migration of cells in opposite directions, these protrusions are elongated in the intercellular space to maintain the cell–cell connection and thus lead to the formation of TNTs. This mechanism is called “cell dislodgement” [101,113]. Macrophages and other cell types can use both mechanisms of TNT formation. Recent reports have shown that a protein known as M-Sec or Tumor Necrosis Factor-α-Induced Protein 2 (TNFAIP2) plays a key role in TNT formation in macrophages [119].

Development of TNTs is enhanced under stress conditions, such as oxidative stress, serum starvation, viral infection, UV irradiation, and high glucose [98]. Several of these stress conditions are often found in the tumor microenvironment, including hypoxia, acidity, low nutrient levels, and metabolically stressed conditions [98,120]. Thus, several indications suggest that TNT formation represents a defensive mechanism that helps to protect cells from death and damage, or to escape from a hostile microenvironment.

An example of such defensive/rescue mechanisms dependent on the TNT-based cellular communication was described by Wang and Gerdes in 2015 [116]. UV-stressed tumor PC12 cells that displayed mitochondrial failure and early apoptosis were co-cultured with healthy PC12 cells. Damaged PC12 cells were able to project TNTs toward healthy PC12 cells. Intact mitochondria were then transferred between cells, thus, reversing the apoptotic status of the UV-stressed PC12 cells.

Several reports have further suggested that TNT formation and cell fusion are tightly associated and can be triggered by similar cues. For instance, cell apoptosis not only triggers formation of TNTs, as described above, but can also enhance cell fusion. In a recent study, increased cell fusion events were detected between early apoptotic breast cancer cells kept in hypoxic conditions and mesenchymal stem/multipotent stromal cells (MSCs) [13]. Upon fusion, cell hybrids possessed higher migratory and survival capacity compared to the parental healthy breast cancer cells.

In a different study using a co-culture model, live-cell time lapse imaging demonstrated that glioma-initiating cells (GICs) could fuse with macrophages [121]. However, before cell fusion occurred, these two types of cells were connected through intercellular membrane protrusions with different structural characteristics. Thin protrusions had the same morphological features as TNTs, in addition to thicker protrusions (called intercellular microtubes that display diameters ranging from 5 to 20 µm) called intercellular microtubes. Nevertheless, TNTs and microtubes facilitated partial cell fusion between GICs and macrophages that connected their cytoplasms. Hence, cell-to-cell interactions between GICs and macrophages ultimately have been shown to lead to spontaneous cell fusion and formation of multinucleated hybrid cells in a subset of the cell population. In fact, incidence of spontaneous cell fusion in this co-culture model was ~3%. Surprisingly, these same hybrid cells were shown to fuse again with each other. Fusion cells generated offspring hybrid cells through symmetrical and asymmetrical division, while a small percentage (<10%) of the population underwent apoptosis [121].

Partial cell fusion due to TNT-based cellular interactions between macrophages and tumor cells could also lead to intravasation into the blood circulation. In fact, recent studies have found that juxtacrine signaling via TNTs and paracrine signaling between tumor cells and macrophages shared certain molecular signaling pathways that favor cell migration and invasion [119,122]. Macrophages interact with tumor cells in the tumor microenvironment, exploiting a well-studied paracrine interaction. In brief, macrophages secrete epidermal growth factor (EGF), which interacts with its target receptor (EGFR) on the surfaces of tumor cells [123]. This binding event activates EGFR signaling, thus, leading to the secretion of colony stimulating factor 1 (CSF-1), which in turn attracts macrophages via CSF-1 receptor (CSF-1R) [123,124]. This paracrine interaction between CSF-1-secreting tumor cells and EGF-secreting macrophages drives the migration of tumor cells and the macrophages toward blood vessels. Interestingly, a recent paper demonstrated that when the ability of macrophages to secrete EGF was inhibited (by blocking metalloproteinase-dependent EGF shedding), only macrophages that were connected to tumor cells via TNTs were still able to promote tumor cell elongation via an EGFR-dependent signaling mechanism [122]. This finding suggests that membrane-bound EGF on the surface of the macrophages might be mobilized along the TNT membrane, where it is able to interact with EGFR on the tumor cell surface. This EGF–EGFR interaction on the surface of TNT might lead to an extended activation of EGFR signaling, since EGFR downregulation via its internalization might be hampered on the TNT membrane. In addition, this paper showed that these partially fused hybrids between macrophages and tumor cells were able to directionally migrate towards endothelial cells [122].

Several recent studies have demonstrated that tumor cells and macrophages undergo partial fusion in the tumor microenvironment through formation of transient heterotypic TNTs. Whether this partial cell fusion leads to permanent cell fusion likely depends on several cues (e.g., biochemical signals or mechanical forces) that regulate tumor microenvironment conditions. Migration of these partially fused hybrid cells toward the blood vessels can also alter the stability of these transient heterotypic membrane connections. In addition, transformative processes, such as epithelial–mesenchymal transition might also contribute to tumor cell migration toward blood vessels. These phenotypic changes in the tumor cells can either trigger permanent fusion with macrophages or lead to the loss of TNTs or any intercellular connection. Thus, upon intravasation into the blood circulation, tumor cells can be detected as circulating hybrid cells or CTCs. As detailed above, circulating hybrid cells are consistently found in patients with cancers of all stages [29,73]. Therefore, further investigations are needed to shed light on the precise molecular mechanisms and series of events that regulate partial and permanent cell fusion.

## 8. Tumor Cell Fusion Leads to Tumor Heterogeneity and Chemoresistance

**Figure 3:**
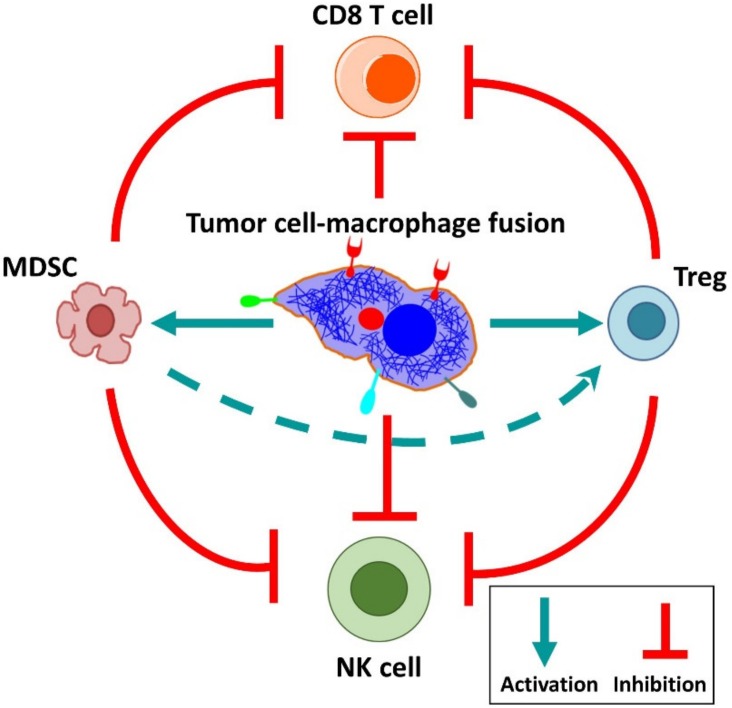
Fusion of tumor cells with macrophages might impact the immune response (Abbreviations: MDSC—myeloid-derived suppressor cell; NK—natural killer cell; Treg—regulatory T cell).

Fusion between tumor cells and other cells (homo- and heterotypic) might represent a key process generating genetic heterogeneity required to metastasize and develop therapy resistance [12,19,34,41,65,125,126]. Heterogeneity caused by the fusion of stromal cells with breast tumor cells leads to mixed gene expression profiles, transition to a carcinoma phenotype [56], and likely contributes to abnormal chromosomal ploidy [125]. Normal intestinal crypt epithelial cells from rats can generate cell fusion that leads to tumor formation with geno-/phenotypic heterogeneity and capacity to form invasive tumors with distinct rates of growth, differentiation, and invasiveness [126]. Hybrids formed between human breast tumor cells MCF-7 were heterogeneous with regard to chemoresistance to doxorubicin [89]; however, hybrids derived from MDA-MB-435 and breast epithelial cells showed altered sensitivity to the phosphoinositide 3-kinase (PI3K) inhibitor Ly294002, as a consequence of differential Rapidly Accelerated Fibrosarcoma-Akt (RAF-AKT) crosstalk between hybrid cells [90]. Further, co-cultivation of murine breast carcinoma and bone marrow-derived cells resulted in significantly increased expression of multi-drug resistance ATP binding cassette (ABC) transporters Abcb1a and Abcb1b [91]. Fusion of hepatocellular cancer and stem cells generated hybrids with significantly increased tumorigenicity and chemoresistance [40]. Finally, it has been suggested that fusion of tumor cells with macrophages might induce immune tolerance towards tumor antigens and immune escape (Figure 3) [5,15,21]. Recognition of peptide–MHC class II by CD4+ T cells stimulates their activation and also mediates interactions between antigen-specific B cells and T helper cells. One tumor-promoting mechanism could be immune response inhibition by tumor antigen presentation via MHC class II receptors present on the macrophage-tumor cell hybrids, directly affecting the CD4+ T cell responses, which could be targeted therapeutically [5,127]. However, although this theory is very attractive it remains to be further studied and experimentally proven.

Taken together, mechanistic studies on the impact of tumor cell fusions on drug resistance might be a successful way to deliver new insights on molecular targets for cancer treatments, including immunotherapies.

## 9. Summary and Perspective for Precision Medicine on Fusion Cells in Cancer

The molecular biology of cancer and the fate of circulating tumor associated cells in the blood remains unclear. A number of reports associate fusions of tumor cells with macrophages and cells of other origins with cancer-promoting features [128]. Circulating cancer-associated cells that have features of fusion are found in many patients with solid cancers and are associated with poor outcome. There is considerable genetic evidence that these cells are a product of cellular and nuclear fusion leading to genetic heterogeneity, and not just a product of phagocytosis with a resulting tumor cell-macrophage hybrid phenotype. Although it might be possible that tumor cells and macrophages use similar mechanisms for cellular fusion as has been described in physiological processes mediated by Syncytins and F proteins, the molecular and biological mechanisms of tumor-cell–macrophage fusions need to be further investigated [31]. The underlying triggers and molecular mechanisms of cellular fusion and the role of intercellular connections like TNTs in cell fusions need to be further studied. The actual fusion sites (primary tumor site, peritumoral microvessels, intravascularly, lymphatic system, and bone marrow) also have to be identified. In the era of evolving cancer immunotherapy, the effects of tumor-cell–macrophage fusion on the immune system deserves to be further explored. The understanding of how tumor cells manipulate the immune response might significantly increase the options for and the response rates of oncological treatments.

Additionally, reliable and repeatable liquid biopsies have a significant potential to have an important clinical impact. This includes cancer screening, diagnostic profiling for personalized treatments, prognostication, detection of minimal residual disease, real-time monitoring of treatment responses, evaluation for therapeutic resistance, and early detection of recurrence. Prospective and large-scale translational studies are needed to further evaluate these avenues, in order to develop strategies for more widespread clinical use. Further, wider improvements in enrichment and characterization techniques for CTC subtypes will enhance our understanding of metastasis biology. Genetic characterization of single tumor cells (fused and non-fused), in the blood through next generation sequencing methods might provide new insights into the complex biology of cancers, with important implications for the clinical management of oncological patients. Expansion of fusion cells through culturing and patient-derived xenograft models will likely play a significant role in studying the tumor biology of these cancer fusion cells and whether the effective inhibition of tumor cell fusion might be formally investigated as a therapeutic target in cancer patients. Taken together, there is convincing evidence to further develop the fascinating field of circulating tumor-associated cells to better understand the biology of metastasis and cell-based liquid biomarkers for the improvement of care in patients with cancer.

## Figures and Tables

**Table 1:** Circulating, cancer-associated cell subtypes (selected).

Description	Phenotype (+: Expression; -: No Expression)
Traditional CTCs [3]	EpCAM+/(cytokeratin)CK+/CD45-, intact DAPI+ nucleus, ≥8 µm
CTC clusters [23]	≥2 EpCAM+/CK+/CD45-/DAPI+ CTCs in aggregate
Apoptotic CTCs [24]	DAPI fluorescence intensity significantly less compared to a CTC Dotted cytoplasmic CK staining pattern (in contrast to filamentous)
CTC debris [24]	EpCAM+/CK+, CD45-, DAPI, <4 µm
EMT CTCs [6,7]	EpCAM+/CK+/EMT+ (e.g., vimentin, N-Cadherin)
Stem-cell CTCs [25,26]	EpCAM+/CK+/CD133+/CD44+/CD24-/ALDH1+
Macrophage-tumor fusion cells (MTFs) [21,22,27,28] Cancer-associated macrophage-like cells (CAMLs) [29]	EpCAM+/CK+/CD14+/CD45+, ≥30 µm Diffuse cytoplasmic CK staining pattern ≥1 DAPI+ nucleus Polymorph cell shape

**Table 2:** Detection of fusion cells in human cancers.

Cancer Type	Fusion	Marker Co-Expression (Detection Method)	References
Breast cancer	Tumor cell-macrophage	CK, CD163, MAC387, DAP12 CD14, CD45 (IHC)	Shabo et al. [20,66,67], Tang et al. [76], Adams et al. [29]
Colorectal cancer	Tumor cell-macrophage	CK, CD14, CD45 (IHC)	Clawson et al. [27], Kaifi et al. [28]
Esophageal cancer	Tumor cell-macrophage	CK, CD14, CD45 (IHC)	Tang et al. [76]
Malignant melanoma	Tumor cell-macrophage	EpCAM, CK, CD14, CD45, CD163, CD204, CD206, ALCAM, MLANA (IHC/IF), BRAF mutations (PCR) Short tandem repeat analysis (PCR)	Clawson et al. [22], Lazova et al. [17]
Multiple myeloma	Tumor cell-osteoclasts	TRACP (IF), specific translocations (FISH)	Andersen et al. [78]
Non-small cell lung cancer	Tumor cell-macrophage	CK, CD14, CD45 (IHC)	Tang et al. [76]
Ovarian cancer	Tumor cell-bone marrow-derived cell	EpCAM, CD45, CA125 (IHC/IF); EpCAM, CD14, CD34, CD44, CD68, CD117, CD133, CD163, CD204, CD206, CA125, CXCR4 (FC)	Ramakrishnan et al. [16]
Pancreatic ductal adenocarcinoma	Tumor cell-macrophage	EpCAM, MIF, ALDH1A1, CD44, CD68, CD163, CD204/MSR1, CD206, CXCR4, S100PBP, Pan-keratin, ZG16B (IHC/IF) CK, CD14, CD45 (IHC)	Clawson et al. [21], Tang et al. [76], Adams et al. [29]
Prostate cancer	Tumor cell-macrophage	CK, CD14, CD45 (IHC)	Tang et al. [76], Adams et al. [29]
Renal cell carcinoma	Tumor cell-bone marrow-derived cell, Tumor cell-macrophage	PCR and blood group alleles, FISH analysis and Y chromosome detection, CK, CD14, CD45 (IHC)	Chakraborty et al. [54], Yilmaz et al. [53], Tang et al. [76]

Abbreviations: IHC—immunohistochemistry; IF—immunofluorescence; PCR–polymerase chain reaction; FISH—fluorescence in situ hybridization; FC—flow cytometry.

**Table 3:** Cancer-associated features associated with tumor cell fusions.

Features	Cancer Type	References
Enhanced migration	Breast cancer Ovarian cancer	Berndt et al. (2013) [85] Ramakrishnan et al. (2013) [16]
Increased invasive and migratory potential	Breast cancer Breast cancer	Noubissi et al. (2015) [13] McArdle et al. (2016) [86]
Metastatic potential after xenografting	Non-small cell lung cancer Malignant melanoma Malignant melanoma Pancreatic ductal adenocarcinoma Hepatocellular carcinoma	Xu et al. (2014) [51] Chakraborty et al. (2000) [65] Clawson et al. (2015) [22] Clawson et al. (2017) [21] Li et al. (2014) [52]
EMT properties	Non-small cell lung cancer Breast cancer	Xu et al. (2014) [51] Zhang et al. (2019) [87]
Stem cell properties	Ovarian Non-small cell lung cancer	Ramakrishnan et al. (2013) [16] Xu et al. (2014) [51]
Genetic evidence of fusion events	Malignant melanoma Endometrial cancer Multiple myeloma Renal cell cancer	Lazova et al. (2013) [17] Varley et al. (2009) [88] Anderson et al. (2007) [78] Yilmaz et al. (2005) [53]
Chemoresistance	Breast cancer Breast cancer Breast cancer Hepatocellular cancer	Yang et al. (2010) [89] Ozel et al. (2012) [90] Nagler et al. (2011) [91] Wang et al. (2015) [40]
Patients’ survival and cancer recurrences	Pancreatic ductal adenocarcinoma Pooled analysis of solid cancers: breast/esophagus/non-small cell lung/prostate/renal cell cancer, pancreatic ductal adenocarcinoma (≥6 fusion cells/7.5 mL whole blood; ≥50 µm cell size)	Gast et al. (2018) [55] Tang et al. (2018) [76]

Abbreviations: IHC/IF—immunohistochemistry/-fluorescence, FISH—fluorescence in situ hybridization, PCR—polymerase chain reaction, FC—flow cytometry, and TRACP—tartrate-resistant acid phosphatase.
